# Evaluation of the plasma quality after filtration

**Published:** 2010

**Authors:** M. Mahmoodian Shooshtari, K. Mousavi Hosseini

**Affiliations:** Research Center, Iranian Blood Transfusion Organization, Tehran, Iran

**Keywords:** Plasma filtration, Blood coagulation factors, Fresh frozen plasma, Blood inhibitor factors

## Abstract

**Background and the purpose of the study:**

The quality of some of the human plasma derived drugs such as coagulation factor VIII and coagulation factor IX which can be used for the treatment of hemophilia A and B, depends on their activity which may be affected by filtration. In this study the quality of plasma with respect to coagulation factors FVII, FVIII, FIX, FV, FXI, Fibrinogen, antithrombin III, anti-plasmin and antitrypsin activities obtained after plasma filtration with CPD (citrate-phosphate-dextrose) using integral filter was evaluated.

**Methods:**

Sixty units of plasma were individually separated from whole blood by centrifugation and immediately filtered by integral filter system. Specific plasma filtration was carried out between 4 and 20 hrs after blood donation. Before filtration, 60 units of non filtered fresh plasmas were kept as control.

Coagulation factors were determined by one-stage clotting assay in an automated system. Antithrombin III activity was determined by immunochrom assay in an automated system. Activity of anti-plasmin was determined by Berichrom α_2_-antiplasmin and antitrypsin activity was assayed with human neutrophil elastase.

**Results:**

The activity of coagulation factors FVIII, FIX, Fibrinogen, FV, and FXI, were not affected by filtration, in all experiments. Filtration only caused negligible change in FVII activity. Antithrombin III, anti-plasmin and antitrypsin activities were not influenced by filtration. Nonfiltrated and filtrated plasma values were not significantly different (P> 0.05).

**Conclusions:**

Plasma filtration dose not result in a measurable impairment of coagulation factors and inhibitors. Although a little changes in FVII activity was observed after filtration, but these filtration-dependent changes apparently have no impact on the therapeutic quality of whole blood- filtered fresh plasma for transfusion.

## INTRODUCTION

Plasma filtration is one of the methods which has been applied widely for leukocyte depletion. The benefits of leukocyte depletion had been demonstrated in the reduction of alloimmunization (1), decrease in allergic and non- haemolytic febrile transfusion reactions (2) and as a possible tool for prevention of transmission of prion infection such as variant Creutzfeld-Jakob Disease (vCJD) by blood components (3). The main disadvantages of leukocyte depletion depend to some technical problems, such as unclear effects of pre-filtration storage and the filtration process by itself (3–5).

The plasma protein of human blood, in particular, is of enormous value to the pharmaceutical industry in the production of therapeutics for the treatment of fibrinogenic, fibrinolytic and coagulation disorders and immunodeficiencies, such as haemophilia, von Willebrand's disease and fibrinogen deficiency. The major therapeutic proteins are: albumin, with some degrees of purity; immune serum globulin, both normal and specific; anti-haemophilic factor such as factor VIII; prothrombin complex comprising factors II, VII, IX, X; and fibrinogen or factor I (6). As it is known for plasmapheresis, an activation of the complement system by the filter material could be expected (7, 8). In addition, a platelet-mediated clotting activation and an activation of neutrophils and monocytes on the filter material could not be excluded (7–9). The present study evaluated the quality of plasma in terms of activities of clotting factors FVII, FVIII, FIX, fibrinogen, FV, FXI, antithrombin III, anti-plasmin, and antitrypsin inhibitor activity after plasma filtration by an integral filter system.

## MATERIALS AND METHODS

### 

#### Processing of blood donations

Sixty units of whole blood were collected randomly from donors in Blood Transfusion Center of Tehran who met national criteria for blood donation. Donors were informed that their plasma would be used for experimental investigations. Fresh plasma was prepared from citrate-phosphate-dextrose (CPD) and it was filtered between 4 and 20 hrs after donation by an integral filter system with polyester fibers in polyvinyl chloride.

Plasma filtration was performed immediately after plasma separation. Samples (40 ml) were taken before and after filtration and spun down. The plasma was collected, aliquoted into 2 ml portions and stored frozen at - 40°C up to 6 weeks until testing. Sixty units of non filtered fresh frozen plasmas were kept as control. All units of filtrated plasma had the quality to yield residual leukocyte count below 1x 106 per unit.

#### Clotting assays

The following parameters were tested with commercially available test kits according to the manufacture's instructions.

Coagulation factors VII, VIII, IX, FV, FXI, and Fibrinogen were determined in one-stage clotting assay (clot-based) in an automated system (STA compact). Antithrombin III was determined by immunochrom assay in an automated system (STA compact). Activity of anti-plasmin was determined by Berichrom α_2_-antiplasmin (Dade Behring, Schwalbach, Germany) and antitrypsin inhibitor activity was assayed with human neutrophil elastase (Serva, Heidelberg, Germany).

For all experiments, FVIII activity was measured immediately after the first thawing of the 2 ml aliquots at 37°C. Student's ttest was used to compare the means of continuous variables. *P*. values <0.05 were considered statistically significant.

## RESULTS AND DISCUSSION

In this investigation a comparison of activities of FVIII, FIX, FV, FXI, Fibrinogen, antithrombin III, anti-plasmin, and antitrypsin in non- filtrated plasma with filtrated plasma from whole blood filtration by filtered bags, did not reveal significant differences. Filtration caused only negligible changes in FVII activity.

Table 1 shows the activities of FVIII, FVII, FIX, FV, FXI, Fibrinogen, antithrombin III, anti-plasmin, and antitrypsin in non- filtrated plasma as control and filtrated plasma.

**Table 1:** Activities of non -filtrated plasma coagulation factor versus filtrate plasma coagulation factors from whole blood filtration by integral filter system.

Parameter (normal range)	Non -filtrated plasma* n=60	Filtrated plasma* n=60	*P*-value
**Fibrinogen mg%**	230	225	0.59
200–400	145–396	130–417	
**FVII**	200	163	0.05
200–400%	159–270	144–178	
**FVIII**	96	84	0.05
200–400%	170–279	205–250	
**FV**	101	91	0.1
70–120%	70–129	88–110	
**FIX**	72	70	0.270
50–150%	66–100	81–88	
**FXI**	105	103	0.29
70–120%	70–115	99–119	

* Figures are presented as median and average.

Non-filtrated and filtrated plasma values are not significantly different (*P*> 0.05).

Specific plasma filtration is a practicable way to reduce the numbers of contaminating leukocyte in FFP from non filtered (10). In this study, it was found that plasma filtration was efficient, yielding plasma with very few residual white blood cells (in all experiments, all WBC counts were <10^4^ /l).

Results of this study by comparison of filtered and nonfiltered FFP showed that storage of samples at - 40°C up to 6 weeks until testing impaired neither the activity of plasma coagulation factors nor of the inhibitors and in most coagulation factors were remained intact. Statistical analysis revealed that non-filtrated and filtrated plasma values are not significantly different (*P>*0.05).

In addition, the activity of coagulation factors FVIII, FIX, Fibrinogen, FV, and FXI, were not affected by filtration, and the inhibitors such as antithrombin III, anti-plasmin and antitrypsin were not influenced by filtration which is in close agreement with earlier reports (7, 11). Filtration had no effect or showed little effects on activities of the coagulation factors and did not increase factor of clotting and fibrinolysis. (7). In their experiments only a strong neutrophil activation was found which depended on the type of filter and whole blood storage conditions (7).

**Table 2:** Activities of non -filtrated plasma inhibitors versus filtrated plasma inhibitors.

Parameter (normal range)	Non -filtrated plasma* n=60	Filtrated plasma* n=60	*P*-value
**antithrombin III**	86	88	0.08
80–120%	71–104	70–110	
**anti-plasmin**	88	90	0.09
80–120%	90–113	86–119	
**antirypsin**	60	71	0.1
57–125%	60–88	58–80	

* Figures are presented as median and average.

Non-filtrated and filtrated plasma values are not significantly different (*P*> 0.05).

Also in another report, plasma filtration appeared to have minimal effects on coagulation parameters (12), where a significant decrease was observed for factor VII in one sample and for factors IX and XI in two samples. Results of another study has shown that if whole blood filtration is performed after storage at room temperature, elastase release will be increased (13). Neutrophil activation has been reported to be involved in the generation of reactive oxygen species, leading to a diminished inhibitor capacity of antitrypsin (13). The neutrophil activation by filter material may be prevented by storage and subsequent filtration at 4°C (13).

In early studies, complement activation was observed with filters from different manufacture's which all had the quality to yield residual leukocyte counts below 1×10^6^ per unit (14). For some filters, postfiltration values were reduced which were obviously due to the adsorptive properties of the filter material (14). From the result of this study it may be concluded that plasma filtration has no effect on the quality of plasma in terms of activity of coagulation factors FVIII, FIX, FV, FXI, and fibrinogen, but decrease activity of FVII which may be due to the characterization of FVII. FVII is the only factor which can be found active by itself in plasma. Also antithrombin III, anti-plasmin and antitrypsin activities were not influenced by filtration.

However, these filtration-dependent changes apparently have no impact on the therapeutic quality of whole bloodfiltered fresh plasma for transfusion.
